# Human Milk Cells and Lipids Conserve Numerous Known and Novel miRNAs, Some of Which Are Differentially Expressed during Lactation

**DOI:** 10.1371/journal.pone.0152610

**Published:** 2016-04-13

**Authors:** Mohammed Alsaweed, Ching Tat Lai, Peter E. Hartmann, Donna T. Geddes, Foteini Kakulas

**Affiliations:** 1 School of Chemistry and Biochemistry, The University of Western Australia, Crawley, Western Australia, Australia; 2 College of Applied Medical Sciences, Majmaah University, Almajmaah, Riyadh, Saudi Arabia; Kunming University of Science and Technology, CHINA

## Abstract

Human milk (HM) is rich in miRNAs, which are thought to contribute to infant protection and development. We used deep sequencing to profile miRNAs in the cell and lipid fractions of HM obtained post-feeding from 10 lactating women in months 2, 4, and 6 postpartum. In both HM fractions, 1,195 mature known miRNAs were identified, which were positively associated with the cell (p = 0.048) and lipid (p = 0.010) content of HM. An additional 5,167 novel miRNA species were predicted, of which 235 were high-confidence miRNAs. HM cells contained more known miRNAs than HM lipids (1,136 and 835 respectively, p<0.001). Although the profile of the novel miRNAs was very different between cells and lipids, with the majority conserved in the cell fraction and being mother-specific, 2/3 of the known miRNAs common between cells and lipids were similarly expressed (p>0.05). Great similarities between the two HM fractions were also found in the profile of the top 20 known miRNAs. These were largely similar also between the three lactation stages examined, as were the total miRNA concentration, and the number and expression of the known miRNAs common between cells and lipids (p>0.05). Yet, approximately a third of all known miRNAs were differentially expressed during the first 6 months of lactation (p<0.05), with more pronounced miRNA upregulation seen in month 4. These findings indicate that although the total miRNA concentration of HM cells and lipids provided to the infant does not change in first 6 months of lactation, the miRNA composition is altered, particularly in month 4 compared to months 2 and 6. This may reflect the remodeling of the gland in response to infant feeding patterns, which usually change after exclusive breastfeeding, suggesting adaptation to the infant’s needs.

## Introduction

miRNAs (or microRNAs) are small non-coding RNA molecules, typically ~22 nucleotide long, that have emerged as crucial regulators of gene expression at the post-transcriptional level [1]. They perform this function by binding on the mRNA during translation to either repress it or cause mRNA degradation [2]. Via their action on the mRNA, they are involved in a wide range of biological processes in mammals, including normal development and disease, targeting cell functions such as cell cycle, proliferation, differentiation, apoptosis, and immune response [3, 4]. These processes are all continuous in the mammary gland during its development, remodelling and maintenance of milk-secretory characteristics in both pregnancy and lactation [5], in which miRNAs are therefore likely to play key roles [6–9].

In addition to the lactating gland itself, miRNAs present in human milk (HM) are thought to have important functions for the infant [7]. HM contains the required nutritional elements for the infant, including carbohydrates, proteins, lipids, and minerals [10], as well as bioactive factors with immunocompetence and developmental functions [11]. At the same time, it contains prokaryotic cells that contribute to healthy gut colonisation in the infant [12], together with different types of maternal eukaryotic cells such as epithelial cells from the lactating mammary tissue, stem cells and immune cells [10, 13, 14], some of which survive the infant’s gastrointestinal tract, potentially conferring immunoprotective and developmental functions [15–17]. It has been postulated that miRNAs, which are abundant in HM, also participate in these functions [7, 18, 19]. Accumulating evidence is suggesting that, similar to plant food-derived miRNA [20, 21], bovine milk miRNA survive the gastrointestinal tract, enter the bloodstream, and exert tissue-specific regulatory functions in the adult [17, 22–24]. These effects are thought to be mediated by the packaging of milk miRNA in exosomes as well as in milk cells and other microvesicles, which protect them from degradation and facilitate their cellular absorption [7, 25–29]. Particularly in the case of HM consumption by the infant, these effects may be extenuated by the fact that the infant gut is more permeable [30] and less acidic than that of the adult [31], further facilitating the survival, absorption and functionality of HM miRNA early in life. Yet, miRNAs as a bioactive component of HM are still largely unexplored.

Recently, it has been shown that HM miRNAs are primarily produced endogenously in the mammary gland, with small contributions from the maternal blood circulation [32]. Moreover, HM is one of the richest sources of miRNA amongst body fluids [33]. Together, these findings suggest lactation-specific regulation and functions of miRNAs for both the mother and the infant. Further, the different fractions of HM have been examined for their miRNA content, with the cell fraction containing more miRNA species than the lipid fraction from the same individuals [32], and than previously profiled skim milk, lipids and milk exosomes [19, 34]. Factors that can influence the miRNA content of HM are poorly understood, with a recent study demonstrating potential effects of milk removal during breastfeeding due to the increase in the cell content of HM post-feeding [33]. Other factors such as the stage of lactation are yet unexplored. Although major macronutrient components of HM, such as fat, protein and lactose, do not systematically change over the course of lactation [35], immunological components such as secretory IgA, lactoferrin and activated leukocytes decrease from colostrum to mature milk, whereas humoral protection increases again later in lactation [14, 36–38]. Further, the status of the lactating epithelium, as reflected by its gene expression, is altered during lactation [39, 40], potentially reflecting progressive differentiation of the gland. Therefore, it is not unlikely that the miRNA expression and content of HM may be subject to changes associated with lactation progression. In this connection, Kosaka et al. (2010) identified a few miRNAs in skimmed human milk, such as miR-181a and miR-155, which were expressed at lower levels after month 6 of lactation [34]. In this study, we sought to determine whether the profile of mature miRNAs in HM cell and lipid fractions changed temporally during the first 6 months of lactation. We used deep sequencing (Solexa) to analyse miRNAs from milk samples collected from lactating women at months 2, 4 and 6 of lactation. The cellular and lipid miRNA composition was compared over the three stages of lactation, and novel miRNA species were predicted using mirdeep.

## Materials and Methods

### Ethics statement and sampling

The study was approved by the Human Research Ethics Committee of The University of Western Australia, and all methods were conducted in accordance with the approved guidelines. All participants provided informed written consent. Healthy breastfeeding mothers (n = 10) were recruited and donated 5 mL of post-feeding milk (at the end of a morning breastfeeding session) longitudinally in months 2 (M2), 4 (M4) and 6 (M6) postpartum (Table 1). Samples were collected aseptically using an electric breast pump (Medela AG, Switzerland), and were immediately transported to the laboratory for analyses.

**Table 1:** Demographic and HM sample characteristics of study participants (n = 10). All values are presented as a range and as mean±standard deviation in brackets, for cell and lipid HM fractions, and in months M2, M4, and M6 of lactation.

	Month 2-Cells (n = 10 samples)	Month 2-Lipids (n = 5 samples)	Month 4-Cells (n = 10 samples)	Month 4-Lipids (n = 5 samples)	Month 6-Cells (n = 10 samples)	Month 6-Lipids (n = 5 samples)
**Maternal age (years)**	36–24 (32.4 ± 3.86)					
**Parity (Number of children)**	1–3 (1.6 ± 0.69)					
**Infant weight (g)**	4,038–6,940 (5,494 ± 831.1)		5,940–8,945 (7,147 ± 900.3)		7,044–9,800 (8,186 ± 945.8)	
**HM fat content (%)**	-	6.0–16.2 (11.3 ± 3.8)	-	8.1–15.6 (11.1 ± 2.9)	-	7.3–13.0 (10.7 ± 2.3)
**HM cell content (/mL milk)**	400,000–2,300,000 (1,299,356 ± 719,432)	-	142,222–4,360,000 (1,234,797 ± 1,246,005)	-	128,889–1,572,000 (666,146 ± 406,928)	-
**Cell Viability (%)**	89–98 (93.284 ± 2.8)	-	88–99 (95.95 ± 3.1)	-	91–98 (94.7 ± 3.1)	-
Total miRNA (NanoDrop)*	0.83–2.10 (1.42 ± 0.49)	0.01–0.09 (0.052 ± 0.03)	0.50–2.10 (1.19 ± 0.47)	0.02–0.09 (0.052 ± 0.05)	0.005–0.05 (1.14 ± 0.57)	0.20–2.10 (0.031 ± 0.02)
Total miRNA (Bioanalyzer)*	0.80–2.00 (1.24 ± 0.35)	0.007–0.039 (0.026 ± 0.01)	0.30–1.50 (0.90 ± 0.38)	0.014–0.036 (0.027 ± 0.01)	0.005–0.037 (1.16 ± 0.50)	0.50–1.90 (0.023 ± 0.01)
**260/280 ratio (NanoDrop)**	1.95–2.08 (2.03 ± 0.04)	2.02–2.15 (2.07 ± 0.05)	1.89–2.08 (2.02 ± 0.06)	2.05–2.08 (2.07 ± 0.01)	1.95–2.08 (2.01 ± 0.05)	2.00–2.09 (2.04 ± 0.04)
**RIN (Bioanalyzer)**	7.70–8.70 (8.39 ± 0.35)	2.80–8.30 (5.56 ± 2.07)	6.70–9.20 (8.22 ± 0.83)	4.20–6.90 (5.72 ± 1.01)	7.60–9.00 (8.28 ± 0.48)	3.30–7.10 (4.36 ± 1.56)
**Known miRNA species**	362–610 (454 ± 82)	285–582 (362 ± 127)	350–639 (460 ± 75)	349–400 (367 ± 19)	358–508 (419 ± 41)	291–378 (349 ± 25)
**Novel miRNA species**	93–156 (122 ± 22)	62–167 (126 ± 43)	87–446 (161 ± 106)	93–272 (178 ± 70)	95–208 (142 ± 42)	81–287 (162 ± 78)

* Total miRNA concentration measured in 1 million cells (μg) or in 1 microliter (μg) of lipids.

### Human milk processing and miRNA extraction

Fat content was measured in fresh whole HM using the Creamatocrit method, as previously described [32, 41]. Subsequently, whole milk was fractionated to obtain purified cell and lipid fractions, as previously described [32]. Briefly, freshly expressed HM was diluted 1:1 with PBS (Gibco, Life Technology, Foster, CA) and centrifuged at 800 *g* for 20 min at 20°C. Purified milk cell and lipid fractions were washed twice separately in PBS at 800 *g* for 5 min at 20°C, and cells were then counted using a haemocytometer. Small RNA sequencing was performed in all n = 30 milk cell samples from the 10 study participants (3 per participant for each of the three lactation stages examined), and in a subgroup of n = 15 lipid samples from 5 study participants (3 per participant for each of the three lactation stages examined). miRNA were extracted from total milk cells and total milk lipids immediately without cryopreservation using the miRNeasy mini Kit (Qiagen, Hilden, Germany) and the miRCURY RNA Isolation-Biofluids Kit (Exiqon, Vedbaek, Denmark) respectively, as previously described [42]. An Agilent Bioanalyzer 2100 instrument (Agilent, CA, USA) with an RNA 6000 Nano Chip kit, and NanoDrop 2000 Spectrophotometer (Wilmington, DE, USA) were used to measure the concentration and purity of the extracted miRNA (Table 1). All miRNA samples were immediately stored at -80°C for small RNA sequencing.

### Small RNA sequencing and bioinformatics analysis

Sequencing libraries were prepared from HM cells and lipids miRNA samples using the Solexa small RNAs protocol as previously described [33, 43, 44]. By using polyacrylamide denture gels for size fractionation, small RNAs ranging 18–30 nucleotides (nt) long were obtained and ligated to 5′-RNA and 3′-RNA adapters, which were transcribed into cDNA. Small RNA primers (Illumina) were added to the cDNA for PCR amplification. After purification of the cDNA products, Illumina HiSeq 2000 platform was used with SE49 lanes to sequence all small RNAs. During data analysis, raw sequences were cleaned of all contaminated and low reads, such as 5' primer contaminants, oversized insertions, and reads shorter than 18 nt (Table A in S1 File), and the quality of reads was also checked in individual samples (n = 45) (S1 Fig). The clean reads of small RNAs were distributed based on the nucleotide size. The vast majority of miRNAs are between 21 and 22 nt, whilst piRNAs are 30 nt, and siRNAs are 24 nt. All clean reads were mapped to the human genome using the SOAP software to analyse their expression and distribution. Thereafter, all of these mapped and cleaned reads were annotated using BLAST to identify different small RNA classes. Therefore, reads were to aligned to Rfam (ftp://sanger.ac.uk/pub/databases/Rfam/) and GenBank (http://blast.ncbi.nlm.nih.gov/) to identify and remove rRNA, tRNA, snRNA, scRNA, snoRNA, and other ncRNAs. Also degraded fragments of mRNAs were removed after the alignment to exons and introns. The remaining reads were mapped to miRBase 21.0 (release August 2014) (http://www.mirbase.org/) using BLAST to determine the human known mature miRNA species. The clean reads that did not map to miRBase and other RNAs but mapped to the human genome, were used for novel miRNA prediction analysis. In this analysis, all unmatched clean reads were uploaded into mirdeep (http://sourceforge.net/projects/mirdeepstar/) by SOAP to explore the stem loop (secondary) structure, the Dicer cleavage site and the minimum free energy of the unannotated small RNAs. Then, base bias on the first position and the nucleotide length on each position were determined for each read for accurate novel miRNA prediction.

### Differential expression analysis

All identified known and novel miRNAs were used for differential expression analysis between different time points (month 2, 4 and 6 of lactation), and between milk cell and lipid fractions. The expression levels of transcript per million (TPM) were obtained using the following normalisation formula: normalised expression = actual miRNA count / total count of clean reads * 1,000,000. Then, fold change was obtained using the following formula: *Fold change = log*_*2*_*(normalised expressed miRNAs from a milk fraction or lactation stage / normalised expressed miRNAs from another milk fraction or lactation stage*. DEGseq, an R package for identifying differentially expressed genes from RNA-seq data, was used to generate scatter plots and determine p-values based on the fold change [45]. miRNAs with p<0.05 were considered to be differentially expressed between different stages of lactation and between HM cells and lipids samples.

### miRNA target prediction, signaling and metabolic functional analysis

Prediction of target genes of the top 20 most highly expressed known and novel miRNAs was done using three different databases/algorithms: targetscan (http://www.targetscan.org/), miRanda (http://www.microrna.org/microrna/home.do), and RNAhybrid (http://bibiserv.techfak.uni-bielefeld.de/rnahybrid). These three databases predict the binding region (miRNA seed region) between the mature miRNA sequence and the mRNA. Furthermore, these targets were classified into different signaling and cellular function ontologies (cellular component, molecular function and biological process) using Gene Ontology (GO) (http://www.geneontology.org/) [46] to investigate the functions of the miRNA target genes. Metabolic and cellular pathways of the identified target genes were also predicted using the Kyoto Encyclopedia of Genes and Genomes (KEGG) (http://www.genome.jp/kegg/) [47].

### Statistical analysis

Microsoft Excel and R Studio Version 0.98.1103 package [48] were used to perform graphical exploration of the data and statistical analyses with linear mixed effects modeling (LME) via nlme [49] and lattice [50] packages. Differences were considered to be significant if p<0.05. Differences in total cell content, fat content and total miRNA content between individual participants, HM cells and lipids, and during the three stages of lactation (M2, M4, and M6) were tested using general linear hypothesis tests and LME. The number of identified miRNA species in individual cell and lipid samples obtained from each mother at each lactation stage was statistically compared using LME models to account for the possibility of individual variation. The verification with LME modelling indicated that there was no random effect due to individual variation in this study. Thus, linear regression modelling was used for the final comparison analysis.

### Availability of supporting data

All raw small RNA sequences are available in the NCBI Gene Expression Omnibus database under accession number GSE75726. Additional information is also included as supplementary files.

## Results

### Cells are richer in miRNA species than lipids in human milk

**Fig 1:**
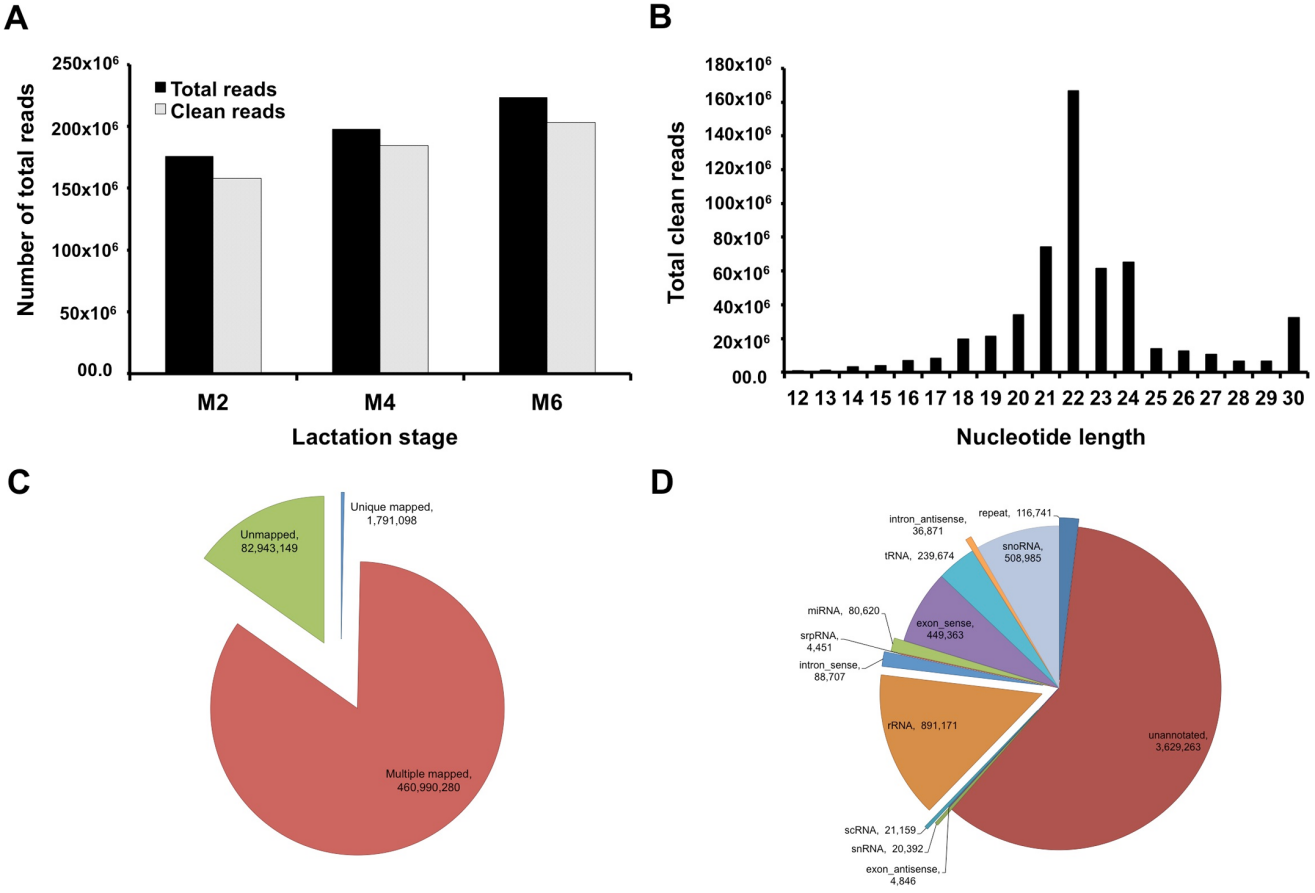
Annotation and filtering analysis of all clean small reads. **(A)** After the filtering analysis of the total reads, the total number of raw generated clean reads compared to the filtered clean reads is presented. **(B)** Total clean reads of small RNAs distributed based on their nucleotide length (12–30 nt). **(C)** Total raw and clean reads for each lactation stage (months 2, 4 and 6). **(D)** Small RNA unique reads categorised into only one RNA type, including miRNAs, using the following priority rule: rRNAetc (in which Genbank > Rfam) > known miRNA > piRNA > repeat > exon > intron3.

Small RNAs (sRNA) including mature miRNAs were sequenced using Illumina HiSeq 2000 (50SE) in HM cell and lipids samples obtained longitudinally from 10 lactating mothers. Filtering analysis was used to determine clean reads, where 545,655,541 clean reads (91.93%; average = 12,125,678.7 reads per sample) were obtained from the total generated 596,988,897 raw reads (Fig 1A). All clean reads were distributed based on their nucleotide (nt) length, in which reads of 22 nt length were the most common (29.13%) (Fig 1B). By using SOAP, 543,933,429 reads were mapped to the human genome to determine the distribution and expression of small RNAs (Fig 1C). All the mapped and clean reads were annotated to the different RNA types in the to Genebank and RFam to identify and remove rRNA, tRNA, snoRNA, snRNA, scRNA and repeat small RNAs (Fig 1D). miRNAs and unannotated total reads were used for known and novel miRNA identification, respectively (Fig 1D). The total reads of 282,555,158 (unique reads 80,620) were matched using BLAST to the miRBase 21.0 to determine human known mature miRNAs. Specifically, higher matched reads were obtained from the cell sample group (n = 30) compared to the lipid sample group (n = 15), with 6,737,566.6 and 5,403,643 average clean reads, respectively (Table B in S1 File).

**Fig 2:**
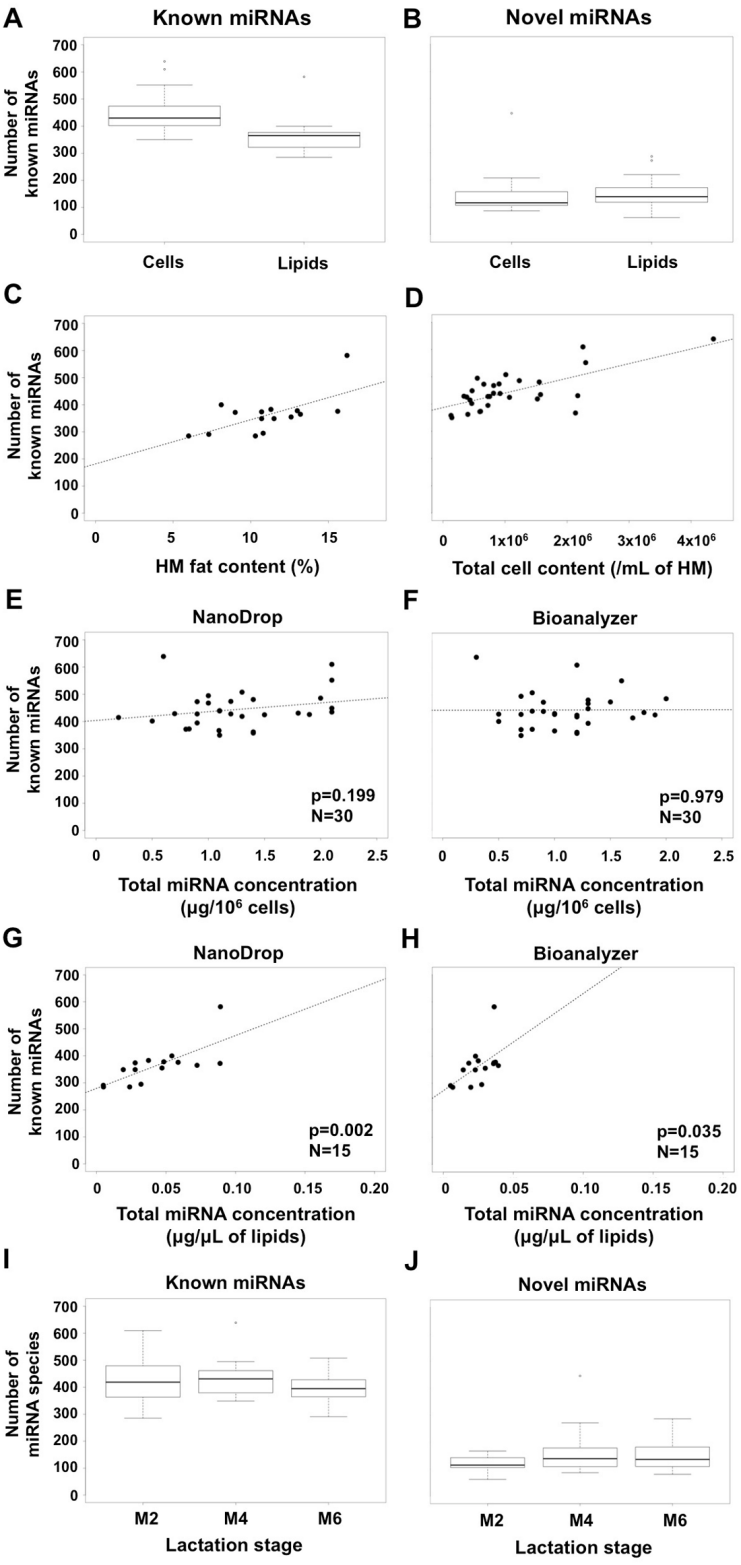
**(A, B)** The number of **(A)** known and **(B)** novel miRNA species in HM cells (n = 30) and lipids (n = 15). **(C)** Relationship between the number of known miRNA species profiled in milk lipids (n = 15) and total fat content. **(D)** Relationship between the number of known miRNA species profiled in milk cells (n = 30) and total cell content. **(E, F)** Relationship between total known miRNA species profiled in HM cells and total HM cell miRNA concentration (μg/10^6^ cells) measured by Nanodrop **(E)** and Bioanalyzer **(F)**. **(G, H)** Relationship between total known miRNA species profiled in HM lipids and total HM lipid miRNA concentration (μg/μL of lipids) measured by Nanodrop **(G)** and Bioanalyzer **(H)**. **(I, J)** Number of **(I)** known and **(J)** novel miRNA species identified in the three stages of lactation examined (n = 10).

1,195 known mature miRNAs were identified in all HM cell and lipid samples (n = 45), of which 1,136 known miRNA species (total matched reads = 202,126,997) and 835 known miRNA species (total matched reads = 81,054,651) were determined in HM cell (n = 30) and lipid (n = 15) samples, respectively (Fig 2A; Table 1; Table C in S1 File), with the HM cell samples conserving significantly more known miRNAs than the lipids samples (p<0.001). The let-7 miRNA family was of the most highly expressed miRNAs in HM, with four let-7 mature miRNAs (let-7f-5p/7a-5p/7i-5p/7e-5p) ranking in the top 20, and other let-7 members (let-7g-5p/7b-5p/7c-5p/7d-5p) expressed at high levels (Table 2; Table C in S1 File). Amongst the identified known miRNAs, most species (776 miRNAs) were commonly observed in both cells and lipids, whereas 360 and 59 known miRNAs were specific to cells and lipids, respectively (Table D in S1 File).

**Table 2:** Top 20 most highly expressed known miRNAs identified in 10 mothers in each lactation stage (month 2, M2; month 4, M4; and month 6, M6) and in the cell and lipid fractions of HM, with the total reads. All the presented miRNAs were identified in all samples in each lactation stage and milk fraction.

miRNA species	M2 (n = 10)	M4 (n = 10)	M6 (n = 10)	Cells (n = 30)	Lipids (n = 15)
**let-7f-5p**	12,401,040	18,703,845	24,056,451	41,882,696	13,278,640
**miR-181a-5p**	9,010,343	8,854,468	8,280,949	17,505,458	8,640,302
**miR-182-5p**	6,920,662	10,029,944	10,727,735	18,396,918	9,281,423
**miR-148a-3p**	6,113,951	6,211,182	6,018,492	13,491,305	4,852,320
**let-7a-5p**	4,390,350	6,082,456	7,290,866	13,054,286	4,709,386
**miR-375**	4,345,215	5,195,961	5,194,769	9,992,213	4,743,732
**miR-22-3p**	3,863,461	4,670,692	4,943,342	8,617,402	4,860,093
**miR-30a-5p**	3,822,156	3,402,934	3,607,107	7,347,279	3,484,918
**miR-141-3p**	2,785,391	2,938,731	3,102,357	6,663,599	2,162,880
**miR-30d-5p**	2,679,770	4,339,844	4,995,894	7,859,146	4,156,362
**miR-146b-5p**	3,899,267	3,910,664	3,667,413	8,119,202.0	3,358,142
**miR-99b-5p**	3,639,837	2,680,089	1,400,374	5,756,657.0	1,963,643
**miR-125a-5p**	2,033,776	1,596,856	1,166,779	3,549,138.0	1,248,273
**miR-10a-5p**	1,958,777	1,679,535	1,151,709	3,403,563.0	1,386,458
**miR-26a-5p**	1,128,571	1,460,349	1,009,074	2,737,027.0	860,967
**miR-21-5p**	1,297,811	1,076,987	641,092	2,524,499.0	491,391
**miR-191-5p**	997,645	1,590,406	817,349	2,402,640.0	1,002,760
**miR-423-5p**	816,934	750,918	665,056	1,789,507.0	443,401
**let-7i-5p**	879,471	800,140	800,269	1,787,511.0	692,369
**miR-143-3p**	162771	1,521,802	80,033	1,739,025.0	25,581

Unannotated reads (total 69,827,843; unique 3,629,263) mapped to sRNAs which corresponded to antisense exons, antisense introns, or intergenic regions on the human genome, and did not map to any other RNAs including known miRNAs, were used to predict novel miRNAs. First, mirdeep was used to predict the secondary structure (stem loop), the Dicer cleavage site and the minimum free energy of the unannotated small RNAs that could be mapped to the genome. Second, all unannotated small RNAs were further assessed to determine the predicted novel miRNA nucleotide base bias on the first position with certain length and on each position. In all HM cell and lipid samples (n = 45), 5,167 novel miRNAs (total reads = 225,937) were predicted using the above criteria. Similar to the known miRNA species, significantly more novel miRNA species were identified in the HM cell samples (n = 30) (3,404 novel miRNAs, with total reads of 461,417) compared to the HM lipid samples (n = 15) (2,072 novel miRNAs, with total reads of 454,673) (p = 0.002) (Fig 2B; Table E in S1 File). Moreover, the number of total miRNA species (known+novel miRNAs) was significantly higher in the HM cell samples (p = 0.005). High-confidence novel miRNAs were determined based on more strict criteria, whereby a novel miRNA identified in ≥3 samples and with total reads >20 was considered as high-confidence novel miRNA. This resulted in 235 high-confidence novel miRNAs (total reads = 814,351) in all HM cell and lipid samples, of which 233 were found in HM cells and 187 in HM lipids (Table F in S1 File). Due to the large number of novel miRNAs predicted in this study, only the top 20 most highly expressed novel miRNAs (Table 3) are shown in S2 Fig, with their secondary structure (hairpin structure of the precursor miRNAs), the Dicer cleavage site, and the minimum free energy. Most of the novel miRNA species found in ≥2 out of the n = 45 samples (276 miRNAs) were specific to HM cells, whilst only 72 novel miRNAs were specific to lipids, and only 81 were commonly seen between cells and lipids (Table G in S1 File). These findings demonstrate that the cell fraction of HM contains more known and novel miRNA species than the lipid fraction.

**Table 3:** Top 20 most highly expressed novel miRNAs identified in the 10 mothers studied in both cell and lipid HM fractions and across the three lactation stages examined (months 2, 4 and 6), with the total reads, and the number of samples that each miRNA was detected in (total samples n = 45).

miRNA species	Mature sequence	Total reads	Nucleotide length (nt)	Number of samples determined in (out of n = 45)
**novel_mir_189**	GCCTGTCTGAGCGTCGCT	751,632	18	13
**novel_mir_2**	ATGTTGGATCAGGACATCC	18,054	19	20
**novel_mir_112**	GACCTCGCCGTCCCGCCCGCC	5,608	21	8
**novel_mir_4**	TCCATATCCCAACCTGTCAGAGT	5,581	23	27
**novel_mir_5**	AATGTGGCTTAGAACATG	3,495	18	27
**novel_mir_392**	GCGCGCCCCCGCCCCGGC	1,520	18	14
**novel_mir_472**	TAGACGGGCTCACATCACC	1,288	19	10
**novel_mir_1905**	TTAGGTCAAGGTGTAGCC	1,242	18	2
**novel_mir_471**	GACCTCGCCGTCCCGCCCGCCG	1,200	22	2
**novel_mir_2797**	GTCGGGGCGGCGGCGGCGGCG	1,104	21	2
**novel_mir_4933**	GTTAGGTCAAGGTGTAGCC	1,080	19	1
**novel_mir_1906**	GCGGCGGCGGCGGCGGGACCG	927	21	2
**novel_mir_1681**	TACGGATCTGGCTTCTGAGA	795	20	4
**novel_mir_397**	TTTAGACGGGCTCACATCACC	766	21	7
**novel_mir_3593**	GCGGCGGCGGCGGCGGCGGGGC	756	22	1
**novel_mir_3**	TCTGCAGGCTGGGATCTGGGAT	753	22	17
**novel_mir_110**	TCTGCAGGCTGGGATCTGGGA	657	21	17
**novel_mir_113**	ACTAGGATTGTGCTTCCCTGG	622	21	23
**novel_mir_197**	TGCACTACAGAACTTTGA	592	18	24
**novel_mir_116**	TGTAAACATCCTTGACTGA	578	19	23

### Human milk miRNAs are positively associated with milk cell and lipid contents

The total cell content of HM was not related to the cellular miRNA concentration (NanoDrop p = 0.309, Bioanalyzer p = 0.705; n = 30). However, a significant positive association between the fat content of HM and the miRNA concentration of the lipid fraction was found (NanoDrop p = 0.019, Bioanalyzer p = 0.003; n = 15). Further, samples with greater fat content contained more known miRNA species in the lipid fraction of HM (p = 0.010, n = 15) (Fig 2C), but the same was not seen for novel miRNAs (p = 0.731). A significant positive association was also seen between the HM cell content (cell number per mL milk) and the number of known and novel miRNA species in HM cells (p = 0.048 and p = 0.021 respectively, n = 30) (Fig 2D). Although in the HM cell samples, total miRNA concentration did not relate to the number of known (NanoDrop p = 0.199, Bioanalyzer p = 0.979; n = 30) or novel miRNA species (NanoDrop p = 0.249, Bioanalyzer p = 0.890; n = 30), in the HM lipid samples there was a positive association with the number of known miRNA species (NanoDrop p<0.001, Bioanalyzer p = 0.052; n = 15), but not the novel miRNAs (NanoDrop p = 0.734, Bioanalyzer p = 0.459; n = 15) (Fig 2E–2H). When the total miRNA concentration in HM cells and lipids was considered together, the positive association between total miRNA concentration and the number of known miRNA species persisted (NanoDrop p<0.001, Bioanalyzer p = 0.005; n = 45). These data indicate that HM samples with high cell and lipid contents contain more miRNAs.

### Cells and lipids of human milk share similar known miRNA profiles, but contain largely different novel miRNAs

**Fig 3:**
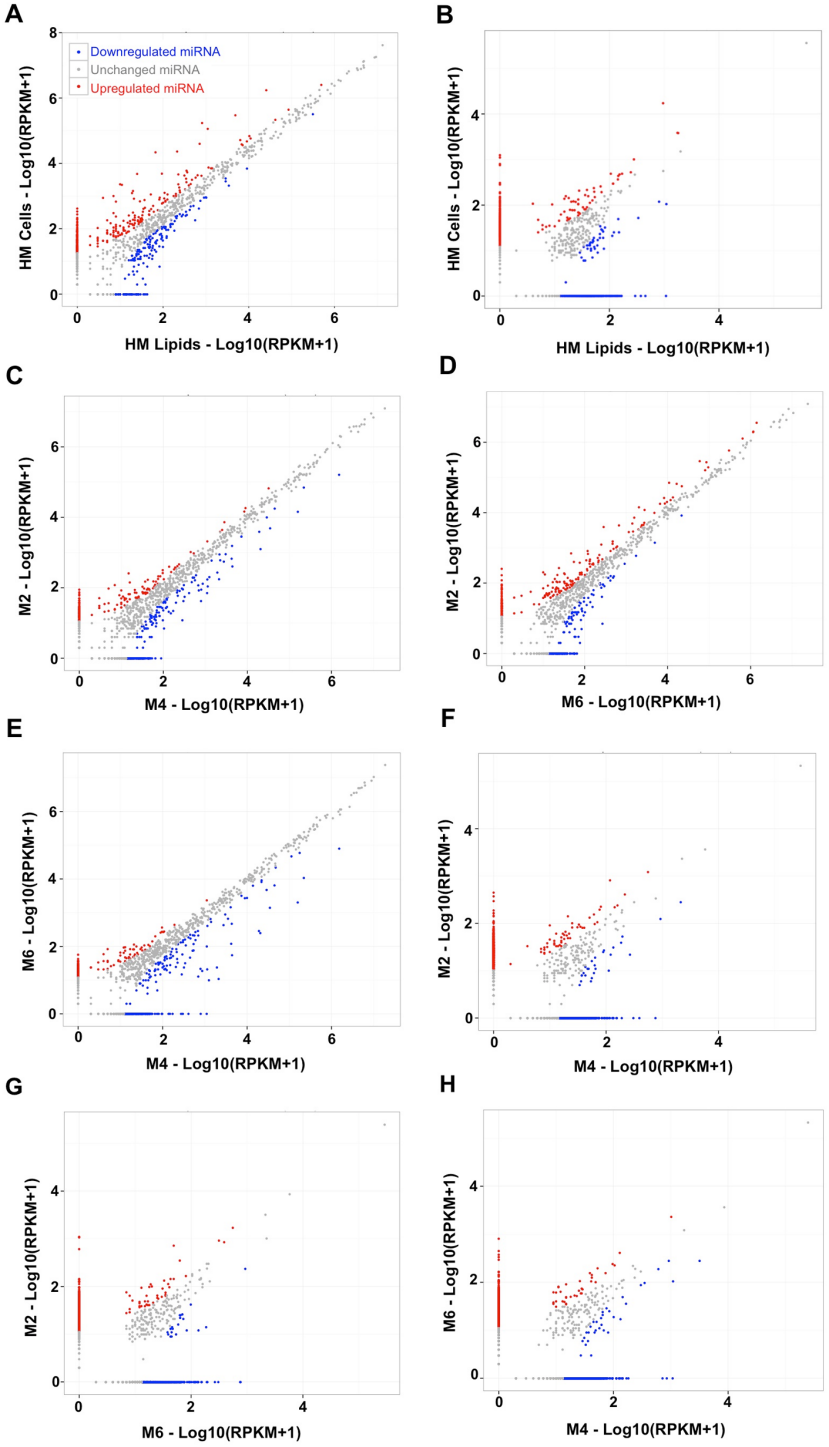
**(A, B)** Scatter plots showing the differentially expressed **(A)** known and **(B)** novel miRNAs in cell and lipid HM samples. **(C, D, E)** Differentially expressed known miRNAs between months M2 and M4 **(C)**, M2 and M6 **(D)**, and M4 and M6 **(E)**. **(F, G, H)** Differentially expressed novel miRNAs between months M2 and M4 **(F)**, M2 and M6 **(G)**, and M4 and M6 **(H)**. Each dot represents an individual miRNA, where red and blue refer to up- and down-regulated miRNAs respectively, and grey dots refer to no change of expression of a given miRNA.

Differential expression analysis was done for the 776 known miRNAs commonly detected between the HM cell and lipid fractions. Of these, 496 miRNAs (63.9%) were expressed at similar levels between cells and lipids (p>0.05) (Fig 3A). The remaining 280 known miRNAs were differentially expressed between HM cells and lipids (p<0.05), with 147 miRNAs found to be upregulated in cells and 133 in lipids (Table H in S1 File). Only miR-21-5p was in the top 20 most highly expressed known miRNAs in cells and lipids, and at the same time upregulated (p<0.001) in HM cells (total reads 41,882,696) compared to lipids (total reads 13,278,640), whilst all other top 19 known miRNAs were similarly expressed between the two milk fractions (S3A Fig). For the novel miRNAs predicted, 309 species were commonly detected in both cell and lipid HM fractions and were thus used for differential expression analysis. Of these, 209 novel miRNAs (67.6%) were expressed at similar levels between the two milk fractions (p>0.05) (Fig 3B), whilst 100 were differentially expressed, with 59 species upregulated in cells and 41 in lipids (p<0.05) (Tables I-J in S1 File). Amongst the top 20 most highly expressed novel miRNAs (present in ≥4 samples), 13 novel miRNAs were upregulated in cells compared to lipids, whilst only 1 miRNA (novel_mir_8) was upregulated in lipids compared to cells (S3B Fig). These results demonstrate that the cell fraction of HM is richer and more variable in novel miRNA species than the lipid fraction, with the majority of novel miRNAs in HM cells being mother-specific. However, approximately 2/3 of all the known and the high-confidence novel miRNAs common between cells and lipids were similarly expressed in the two HM fractions.

### Human milk miRNA expression profiles change with lactation

The overall HM cell and fat contents were not significantly different between the three stages of lactation examined (M2, M4, and M6) (p = 0.109; p = 0.691, respectively). In keeping with this, the total miRNA concentration extracted from HM cells or lipids and measured by NanoDrop and Bioanalyzer did not differ between the stages of lactation (p = 0.226 and p = 0.683 respectively for HM cells; p = 0.452 and p = 0.661 respectively for HM lipids). Overall, the total number of known and novel miRNA species was not different between the three stages of lactation amongst the participants (p = 0.285 and p = 0.355, respectively) (Fig 2I and 2J). Specifically, in M2 951 known (total reads 83,832,174) and 1,601 novel (total reads 260,148) miRNAs in both milk cells and lipids were identified, whilst 996 known (total reads 99,378,288) and 2,181 novel (total reads 75,008) miRNAs were determined in M4, and 867 known (total reads 99,971,186) and 1,950 novel (total reads 311,005) miRNAs in M6. Although the number of novel miRNAs was largely different between the three lactation stages, this was not statistically significant (p>0.05) due to the great variability seen in the novel miRNA composition of each HM sample and the presence of many of these novel miRNAs in single samples.

**Fig 4:**
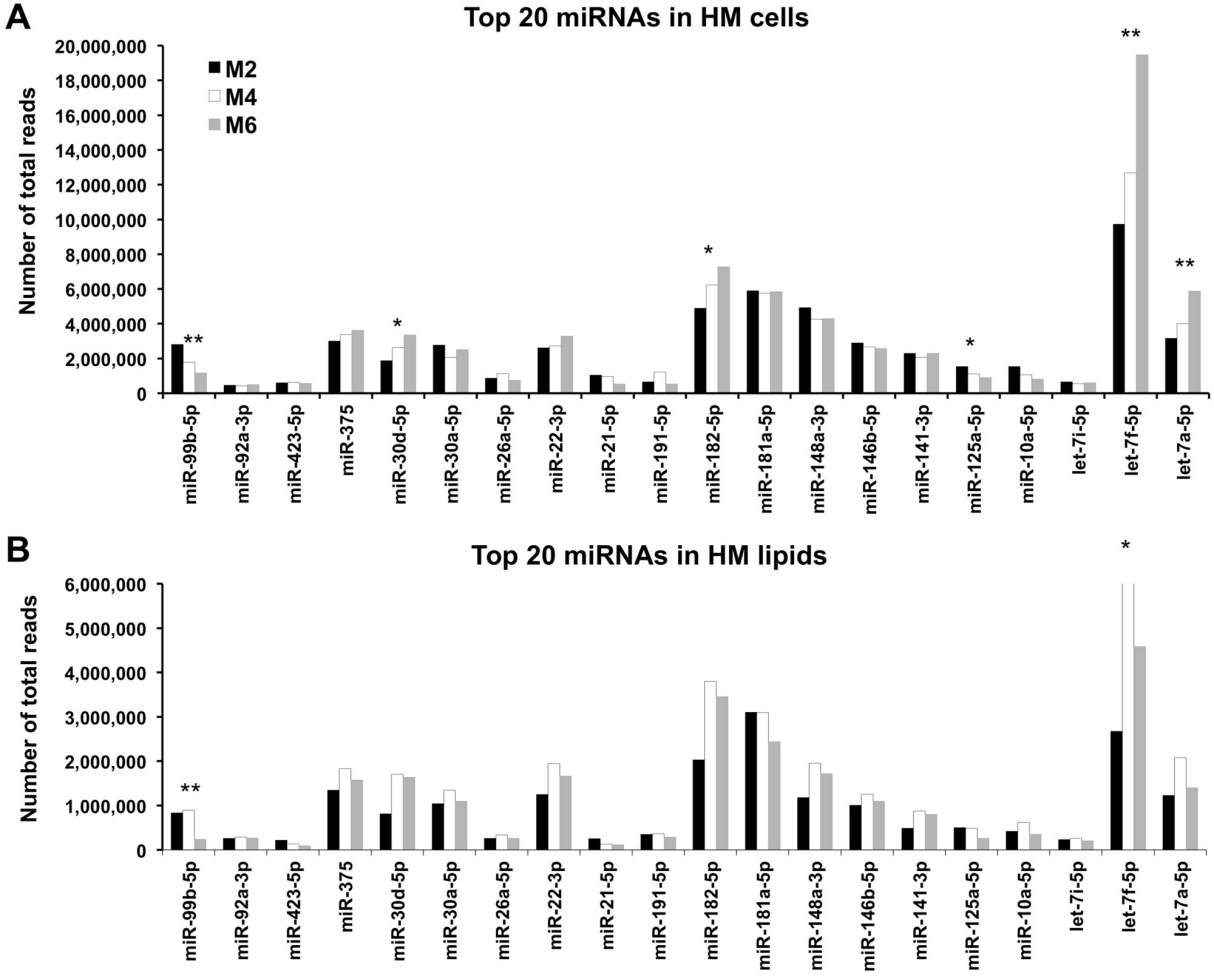
Top 20 known miRNAs in HM cells (A) and lipids (B) in each lactation stage (months 2, 4 and 6 postpartum). The contribution of the top 20 known miRNAs in the cell and lipid fractions was 88.4% and 88.2%, respectively, compared to all identified miRNAs in each fraction. *: p<0.05, **: p<0.01.

The mean total contribution of the top 20 most highly expressed known miRNAs in the cell and lipid HM fractions together was 88.8 ± 1.4% mean ± S.D. (range 87.9–90.3; specifically for each month: M2 87.9%, M4 88.0%, M6 90.3%). Of this, 19.2 ± 4.6% mean ± S.D. (range 14.8–24.1; specifically for each month M2 14.8%, M4 24.1%, M6 18.8%) of copies were related to the most abundant miRNA in HM, let-7f-5p. In the HM cell and lipid fractions separately, the top 20 known miRNAs contributed 88.19 ± 3.2% and 89.4 ± 6.2% mean ± S.D., respectively, of all identified miRNAs in each milk fraction. In particular, the highest expressed miRNA let-7f-5p contributed 23.1 ± 5.7% mean ± S.D. in HM cells, and 17.9 ± 3.6% mean ± S.D. in HM lipids (Fig 4).

**Fig 5:**
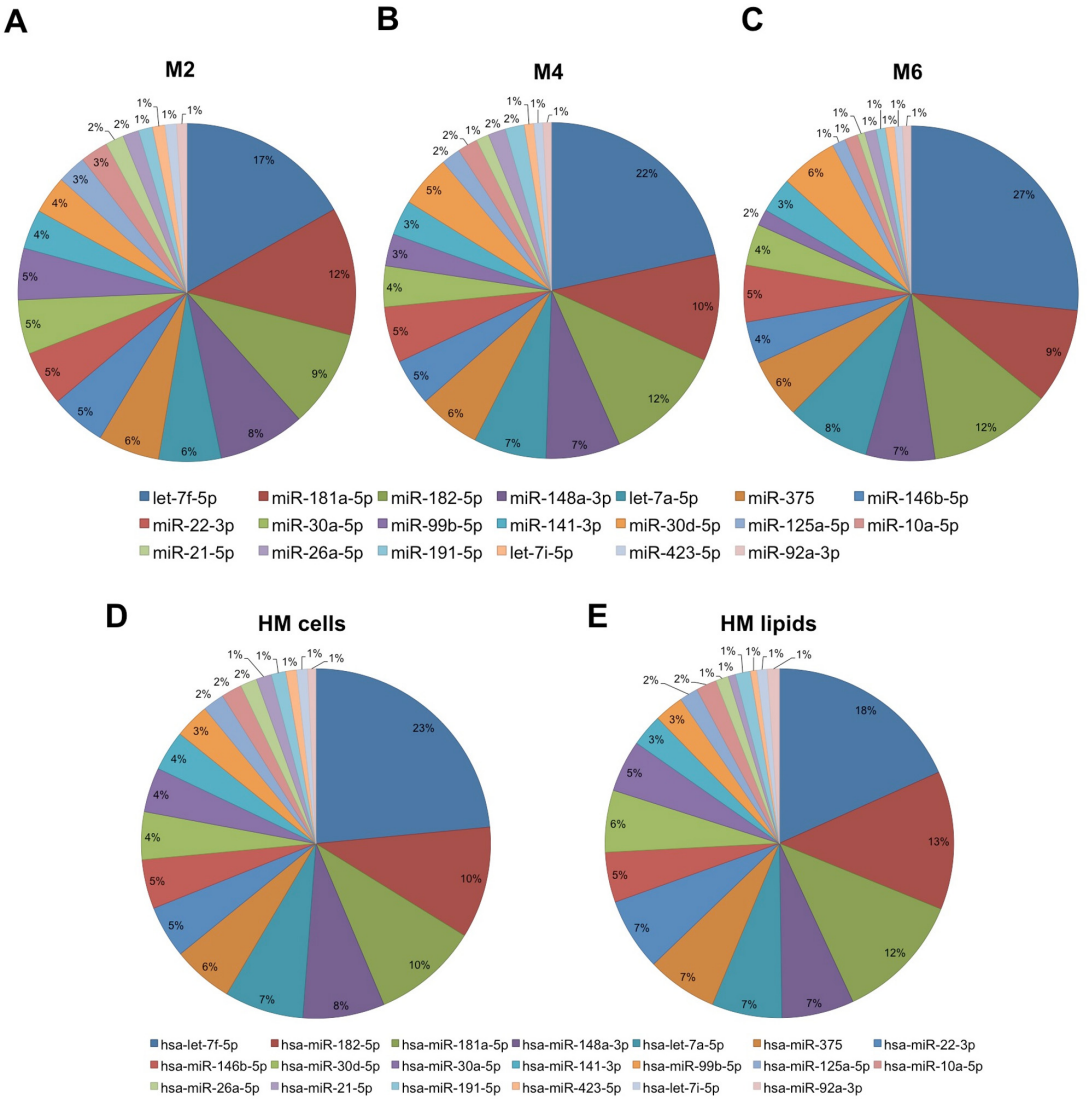
Distribution percentage of the top 20 most highly expressed known miRNA species in each stage of lactation examined (months 2, 4 and 6; n = 10 in each month) (A-C), and in HM cells (n = 30) (D) and lipids (E) (n = 15). The top 20 most highly expressed known miRNAs contributed 88.8±1.4% of all identified known miRNAs.

**Fig 6:**
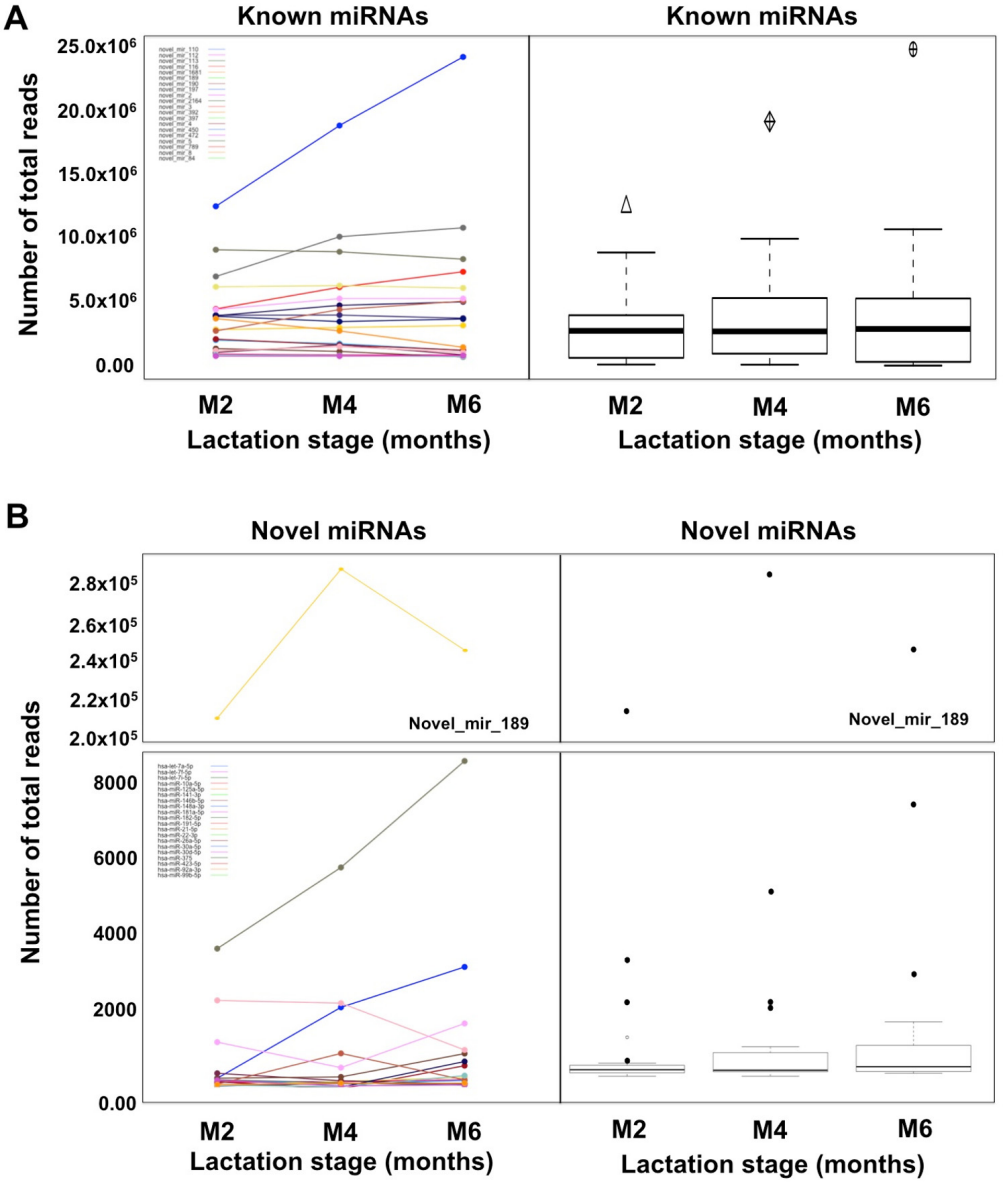
Comparison of expression of the top 20 most highly and commonly expressed known (A) and the 20 most highly expressed novel (B) miRNAs in the cell and lipid fractions of HM considered together, during the three stages of lactation examined (months 2, 4 and 6). Due to the variability in the total reads between novel miRNAs **(B)**, novel_mir_189 is presented separately from the rest. From the top 20 known and novel miRNA species, only the most highly expressed known miRNA let-7f-5p was expressed at higher levels from month 2 to month 6 (p<0.01).

Differential expression analysis between the three stages of lactation for HM cells and lipids together included most of the expressed known miRNAs, since they were identified in all stages of lactation (808 known miRNAs were common between M2 and M4; 746 known miRNAs were common between M2 and M6; and 764 known miRNAs were common between M4 and M6) (Fig 3C–3E). When the cell and lipid HM fractions were considered together, most known miRNAs were similarly expressed between the three stages of lactation amongst participants, whereby 75.5% (610/808) and 71.6% (534/746) of miRNAs did not differ in M2 compared to either M4 or M6 respectively (p>0.05), and 66.6% (509/764) miRNAs were expressed similarly between M4 and M6 (p>0.05) (Table H in S1 File). Of the differentially expressed miRNAs, more known miRNAs were upregulated in M4 compared to M2 and M6 (112/198, 56.6%, and 158/174, 90.8% respectively). 12 of the top 20 known miRNAs in HM cells and lipids together were similarly expressed, based on total raw reads, between the three stages of lactation (p>0.05) (Figs 4 and 5; Table K in S1 File). The majority of upregulated known miRNAs out of the top 20 (4 miRNAs) were seen in M2 compared to M6. Interestingly, let-7f-5p was the most highly expressed miRNA in HM and dramatically increased from M2 to M6 (p<0.001) (Fig 6A; S3C Fig).

In contrast to the known miRNA species, the vast majority of novel miRNAs (89.3%) were identified in few samples at low expression levels. Therefore, the novel miRNA species predicted appeared to be highly variable both within and between individuals, in both the cell and lipid HM fractions and throughout the first 6 months of lactation. When the cells and lipid HM fractions were considered together, most novel miRNAs common in all three lactation stages were similarly expressed between the three stages of lactation (p>0.05) (66.9%, 67.3% and 74.4% between M2 and M4, M2 and M6, and M4 and M6, respectively) (p<0.05) (Fig 3F–3H; Table I in S1 File). When the two milk fractions were considered separately, from the top 20 most highly expressed novel miRNAs only 9 were commonly determined in M2, M4 and M6 in HM cells. Amongst these 9 miRNAs, novel_mir_189 contributed 92.0 ± 1.95% mean ± standard deviation (range 93.8–98.9%). Similarly, only 7 novel miRNAs were commonly identified in M2, M4, M6 in the HM lipid fraction, with the same novel miRNA (novel_mir_189) contributing 98.1 ± 1.5% mean ± standard deviation (range 96.4–99.1%) (Table K in S1 File).

Out of the 235 high-confidence novel miRNAs, 36.9%, 39.2% and 33.1% were differentially expressed between M2 and M4, M2 and M6, and M4 and M6, respectively (p<0.05) (Table J in S1 File). Most of the differentially expressed miRNAs were upregulated between the three lactation stages (64.4% M2 to M4; 53.5% M2 to M6; and 67.9% M4 to M6). The expression patterns of the top 20 most highly expressed novel miRNAs (present in ≥4 samples) in HM cell and lipid samples together were analysed, with most changes occurring in the first 4 months of lactation and the fewest differentially expressed novel miRNAs out of the top 20 observed from M4 to M6 (6 versus 10 in M2 to M4, and M2 to M6). 7 and 3 novel miRNAs were up- and down-regulated respectively in M2 compared to M4; 4 and 6 novel miRNAs were up- and down-regulated respectively in M2 compared to M6; and 5 and 1 novel miRNAs were up- and down-regulated respectively in M4 compared to M6 (p<0.05) (Table K in S1 File). Interestingly, the most highly expressed novel miRNA (novel_mir_189) was enriched in M4 compared to M2 and M6 (Fig 6B; S3D Fig).

### Gene targets and functional analysis of miRNAs in human milk cells and lipids

To determine the main functions of the top 20 most highly expressed miRNAs in HM, target genes were first predicted using different databases (TargetScan RNAhybird and miRanda). These were effectively the same between the cell and lipid fractions of HM because the top 20 most highly expressed known miRNAs were very similar between the two fractions (Fig 5D and 5E). Differences were found for the top 20 novel miRNAs, which differed both between and within participants. The target genes of known and novel miRNAs were predicted using as the 2–7 binding site between the miRNA and mRNA, which is commonly known as the miRNA seed region. The total common number of the gene targets identified in these databases for the top 20 most highly expressed known miRNAs during the first 6 months of lactation was almost identical, where 50,308 gene targets were determined in HM cells and 50,301 in HM lipids (Table L in S1 File). Similarly, the total common number of the gene targets identified in these databases for the top 20 most highly expressed novel miRNAs that were common amongst all participants during the first 6 months of lactation was 50,313 gene targets in HM cells and 50,311 in HM lipids (Table M in S1 File). These gene targets were used to investigate the predicted roles of these miRNAs using GO and KEGG. Most of identified gene targets were involved in numerous essential biological processes including programmed cell death, cell-to-cell communication, cell adhesion, peptide transport, nervous and immune system development, and metabolic processes (Table N in S1 File). More than 600 gene targets were involved in the immune response to various infectious diseases, in particular of bacterial origin. Further, ~500 genes participated in the development of the immune system (Table N in S1 File). Transporting molecules, such as proteins, into and out of cells was one of the most common functions of the genes targeted and regulated by the highly expressed miRNAs in HM. Numerous miRNA gene targets were also involved in growth factor receptor synthesis. Different metabolic processes were also targeted, including fat digestion and absorption and gluconeogenesis pathways (Table O in S1 File).

## Discussion

Human milk (HM) has been well characterised in the past few centuries as the optimal food source for infants, especially in the first 6 months postpartum [51]. It contains all the required nutritional elements to nourish the infant, including fat, carbohydrates, proteins, and vitamins [52], with recent research advances revealing protective and developmental functions of HM conferred by an array of molecular and cellular components, which include miRNA [5, 7, 10, 13]. These small non-coding RNA molecules have been profiled in the milk of different mammals, with HM found to be one of the richest sources of miRNAs in humans [32, 33, 53]. HM miRNAs are protected within cells and exosomes [19, 28, 29], and are likely transferred to different infant tissues exerting regulatory functions [7, 22, 24, 54]. Further to their roles in the breastfed infant, we have recently identified several HM cell miRNAs that are endogenously synthesized in the breast and are involved in the synthesis and regulation of milk components such as triacylglycerol, fatty acids, lactose, and others [32, 33], supporting the involvement of miRNA in the normal function of the lactating mammary gland [7]. Here, we performed a first comparison of the miRNA profiles of the cell and lipid fractions of HM using Solexa sequencing, and examined how they vary temporally in the first 6 months postpartum. The high number of clean small RNA reads (~546M), the high quality and purification levels of the extracted miRNAs (91.9%) [42], and the use of the most updated miRBase (21.0) resulted in the discovery of numerous putative miRNAs that exceeded those reported in previous investigations [18, 43]. Of these, 1,195 were known mature miRNAs, and 5,167 were novel predicted miRNAs, of which 235 were high-confidence miRNAs. This is in agreement with previous reports on porcine milk exosomes, which contain more novel predicted miRNAs than known miRNAs [55]. The cell fraction of HM contained more known miRNAs than the lipid fraction, and had very different novel miRNA profiles, which varied greatly amongst mothers. 36.1% and 80% of known and novel miRNAs, respectively, were differentially expressed between the two milk fractions. However, most of the highly expressed known miRNAs were commonly found in both cell and lipid HM fractions, suggesting that these miRNAs originate from the lactocyte [32], and they are protected within the cells and fat globules to resist digestion in the infant’s gastrointestinal tract [24]. These findings confirm previous postulations [32, 33], and emphasize that cells are the richest in miRNA fraction of HM. Although the total miRNA concentration of HM cells and lipids did not systematically change in the first 6 months of lactation, changes in miRNA composition and expression levels were observed for some miRNAs, particularly in month 4 compared to months 2 and 6 postpartum, potentially reflecting adaptation to infant needs, which change after exclusive breastfeeding.

Increasing the number of reads and using optimised miRNA extraction protocols [42] contributed to the discovery of a significantly higher number of novel miRNAs (5,167, of which 235 were of high-confidence), compared to previous studies, which predicted 21 and 622 novel miRNAs in HM and tammar wallaby milk, respectively [18, 56]. Similar to known miRNAs, milk cells were richer in novel miRNAs (3,404) compared to lipids (2,072). These novel miRNAs were subjected to multiple strict criteria to be considered true novel miRNAs (Table F in S1 File). As expected, all of these miRNAs were present at very low expression levels, suggesting that potentially a large number of miRNAs remain to be discovered as detection methods improve.

The total miRNA concentration of the HM cells and lipids was positively associated with the number of both known and novel miRNA species (Fig 2C and 2D). Moreover, HM fat content was positively related to the number of known miRNA species, and HM cell content was positively related to the number of both known and novel miRNA species (Fig 2G and 2H). Therefore, the total fat and cell contents of HM of a given mother can be indicative of the miRNA concentration and number of species of these milk fractions, as they are also indicative of breast fullness [10, 41, 57]. Indeed, emptier breasts (post-feeding) contain milk that is richer in both fat and cells than fuller breasts (pre-feeding) [10, 41, 57], and appear to also contain more miRNAs associated with the cell and lipid fractions [33]. This supports ‘feeding on demand’ practices, which ensure provision to the infant of not only variable amounts of fat and cells in response to the specific feeding patterns of each infant, but also of the full spectrum of miRNAs of HM.

Of the top 20 most highly expressed known miRNAs identified in this study, very few were differentially expressed between the cell and lipid HM fractions and the three lactation stages (Figs 3A and 5A–5C; Table K in S1 File), indicating similar profiles both between and within individuals and suggesting potentially important functions for these highly conserved miRNAs. This, together with previous reports supporting that most milk miRNAs primarily originate from the lactating mammary gland [32], suggests that some of these highly expressed and conserved during lactation HM miRNAs could be related to the continuous remodelling of the lactating breast associated with cell turnover and milk production, which is known to remain consistent during established lactation. Thus, they could potentially be used as indicators of lactation performance and any pathologies of the gland during the breastfeeding period. Further, it can be postulated that some of these miRNAs, which are known to be involved in numerous fundamental events of tissue development, play important regulatory functions in the rapid development of different tissues and organs of the infant that occurs early in life. Milk exosomes have been found to protect milk miRNAs from harsh digestive conditions, such RNase and high PH [19, 34], and facilitate transfer of milk miRNAs into the bloodstream and host cells via endocytosis [24]. Recently, Hassiotou et al. (2015) provided evidence that native milk stem cells migrate and integrate into the neonate’s stomach, thymus, liver, pancreas, spleen, and brain [54], whereas previous reports have shown this for milk immune cells [16, 17, 58]. It has been suggested that in addition to exosomes, milk cells and fat globules may provide a similar protection to miRNAs during breastfeeding to further facilitate their transport into the bloodstream and their functionality in the infant [7].

Consistent with previous studies [33], 496 known miRNAs (63.9% of the total known miRNAs), were similarly expressed between the two HM fractions (Table I in S1 File). Apart from miR-21-5p, the top 19 highly expressed known miRNAs did not differ between the two HM fractions. These similarities in the profiles of the top 20 most highly expressed known miRNAs between HM cells and lipids suggest that the known miRNAs of the two fractions are mainly reflective of the lactocyte, from which HM fat originates [59]. Still, approximately a third of known miRNAs were differentially expressed between the two fractions (Table I in S1 File). Moreover, the novel miRNAs were mother-specific, with very different profiles between the two HM fractions (Table G in S1 File). This suggests that the cell fraction of milk is more appropriate for novel miRNA discovery studies. Of the top 20 most highly expressed novel miRNAs, 13 were upregulated in cells and 1 in lipids. These differentially expressed known and novel miRNAs between HM cells and lipids need to be further investigated for any specific roles in the lactating breast and/or the infant, and they likely reflect the fact that the miRNA concentration of the lipid fraction is representative of the lactocytes, whilst that of the cell fraction of not only the lactocytes, but also the whole cellular hierarchy of the lactating epithelium. This is also reflected in the absence of a relationship between the miRNA concentration of the cells and the HM cell content or the number of known and novel miRNA species (p>0.05), contrary to the positive association between the miRNA concentration of the lipids and the HM fat content or the number of known miRNA species (p<0.05). The above further indicate that the miRNA content and composition of the HM fat represents specifically the lactocyte, whereas that of the HM cells represents a range of different cell types, with different transcription and miRNA synthesis rates, and is thus more variable.

The HM fat and cell contents and the miRNA concentration of cells and lipids did not change in the first 6 months of lactation. Yet, approximately a third of all known miRNAs were differentially expressed across this period. Although the abundant HM miRNAs that are conserved at similar expression levels may play important functions in the breast and/or infant that are consistent during the first 6 months postpartum, those miRNAs that are differentially regulated may reflect the remodelling of the mammary gland in response to changing infant feeding patterns, which usually occur in the transition from exclusive to non-exclusive breastfeeding. Similar changes in expression of certain milk miRNAs with the stage of lactation were previously reported in other mammals such as the bovine [23] and porcine [60].

HM-enriched miRNAs of the let-7 family, in particular let-7f-5p which was the miRNA with the highest expression here (Fig 4A and 4B; Table C in S1 File) and in previous studies [33], gradually increased in the first 6 months postpartum (Fig 6A), suggesting significant regulatory functions. Further, most of the let-7 miRNA family members have also been found to be in the most highly expressed miRNAs in cow’s skim milk [23]. Gene target analysis using TargetScan (release 7.0) revealed more than 100 genes that can be targeted by let-7f-5p. GO analysis on these targets showed several cellular metabolic processes regulated by let-7f-5p, including protein, carbohydrate, and triglyceride synthesis. This abundant HM miRNA has also been found to play a critical role in tissue development [61], especially of the nervous system [62]. Moreover, HM highly expressed miR-22-3p was identified to regulate T lymphocyte differentiation and development [63]. Other highly expressed HM miRNAs, such as miR-182-5p/181a-5p, were also found to play different roles in immune response and immune cell differentiation. The latter miRNA (miR-181a-5p) has been found at high levels in thymocytes [64], promoting their differentiation into mature T lymphocytes that respond to foreign pathogens [65]. Interestingly, it has been recently reported using a murine model that milk stem cells migrate in large numbers to the neonatal thymus, where they integrate and differentiate into thymocytes [54], a process that may be further facilitated by milk cellular miRNA such as miR-181a-5p. In addition, the thymus of breastfed infants is known to be larger in size compared to the thymus of formula-fed infants [66], further suggesting involvement of HM miRNA in the maturation, development and function of the infant’s thymus.

Other highly expressed HM miRNAs were identified with involvement in numerous biological functions and potential significance for the infant (Tables N-Q in S1 File). For example, HM miR-375 acts in pancreatic islets and is required for normal glucose homeostasis in response to insulin increase [67]. HM miR-148a-3p regulates the DNMT1 enzyme, participating in liver development, and also acts as a tumour suppressor [68]. Some members of the let-7 family, which are abundant in HM, particularly let-7f-5p, are known to play important roles in various biological functions, such as controlling cell differentiation early in development [69] and influencing growth and development [61, 70]. In addition to the known miRNAs, the top 20 novel miRNAs in HM cells and lipids were found to be important regulators of cell growth and immune system development, in particular hematopoietic or lymphoid organ development and somatic diversification of immune receptors (Tables P-Q in S1 File).

## Conclusion

Our findings highlight HM as one of the richest sources of miRNAs in the human body. Numerous known miRNAs were identified here for the first time in HM cells and lipids, as well as numerous high-confidence novel miRNAs, which may play significant regulatory functions in the lactating mammary gland and/or the breastfed infant. HM cells, exosomes and fat globules are thought to act as protective vehicles transferring these miRNAs to the infant’s bloodstream and different tissues, with particular interest in HM cells. These appear to harbour the greatest number of miRNAs in HM, and have recently been shown to survive the infant’s gastrointestinal tract and be distributed and integrated into various infant tissues [54]. Certain highly expressed HM miRNAs involved in development, growth and metabolic processes are conserved in the first 6 months of lactation, potentially participating in the maintenance of milk production during established lactation and/or the development of the infant. The variation seen in the composition of approximately a third of HM miRNAs across this period, together with the mother-specific profiles of novel miRNAs, suggests adaptation to infant needs early in life.

## Supporting Information

S1 Fig Quality control of small RNA sequencing. Reads quality control in each sample (n = 45) after cleaning, where miRNA length is considered between 18–24 nt.
(TIFF)

S2 Fig Top 20 most highly expressed novel miRNA hairpin structures using mirdeep software with their nucleotide (nt) lengths and chromosome locations. (TIFF)

S3 Fig Heat maps demonstrating the relationship and expression patterns of the top 20 most highly expressed known (A) and novel (B) miRNAs between cells and lipids, and the top 20 known (C) and novel (D) miRNAs between the three stages of lactation. The expression levels were analysed hierarchically by clustering the top 20 miRNAs, where the blue colour shows the downregulated miRNAs, whilst the red colour shows the upregulated miRNAs.
(TIFF)

S1 File Tables A-Q. **Table A**. Cleaning of raw reads in each sample for all HM fractions and lactation stages. **Table B**. The number of total clean and matched reads to miRBase obtained from cell (n = 30) and lipid (n = 15) samples. **Table C**. The list of the identified known miRNAs and their total reads in all samples (n = 45), cell samples (n = 30), and lipid samples (n = 15). **Table D**. The common and specific known miRNA species between HM cells and lipids. **Table E**. The list of all predicted novel miRNAs and their total reads in all samples (n = 45). **Table F**. High-confidence predicted novel miRNAs (≥3 samples with total reads of >20) in all samples (n = 45). **Table G**. The common and specific novel miRNA species (present in ≥2 samples) between HM cells and lipids. **Table H**. Differentially expressed known miRNAs (p<0.05) between cells and lipids, M2 and M4 of lactation, M2 and M6 of lactation, and M4 and M6 of lactation. **Table I**. Differentially expressed novel miRNAs (p<0.05) between cells and lipids, M2 and M4 of lactation, M2 and M6 of lactation, and M4 and M6 of lactation. **Table J**. Differentially expressed high-confidence novel miRNAs (p<0.05) between M2 and M4 of lactation, M2 and M6 of lactation, and M4 and M6 of lactation. **Table K**. Differential expression analysis for the top 20 known (A) and novel (B) miRNAs that were present in ≥4 samples (p<0.05) between cells and lipids, M2 and M4 of lactation, M2 and M6 of lactation, and M4 and M6 of lactation. **Table L**. Common gene targets of the top 20 most highly expressed known miRNAs in the cell samples (n = 30), lipid samples (n = 15), M2 of lactation (n = 10), M4 of lactation (n = 10), and M6 of lactation (N = 10) using TargetScan, RNAhybrid and miRandna. **Table M**. Common gene targets of the top 20 most highly expressed novel miRNAs in the cell samples (n = 30), lipid samples (n = 15), M2 of lactation (n = 10), M4 of lactation (n = 10), and M6 of lactation (N = 10) using TargetScan, RNAhybrid and miRandna. **Table N**. Gene targets of the top 20 most highly expressed known miRNAs classified into different functions using Gene Ontology (GO). **Table O**. KEGG pathways identified for gene targets of the top 20 most highly expressed known miRNAs. **Table P**. Gene targets of the top 20 most highly expressed novel miRNAs classified into different functions using Gene Ontology (GO). **Table Q**. KEGG pathways identified for gene targets of the top 20 most highly expressed novel miRNAs.
(XLSX)
