# Supplementary figures and images for: Human Milk Cells and Lipids Conserve Numerous Known and Novel miRNAs, Some of Which Are Differentially Expressed during Lactation
Source: PLoS One. 2016 Apr 13;11(4):e0152610. doi: 10.1371/journal.pone.0152610 (PMC4830559; doi:10.1371/journal.pone.0152610)

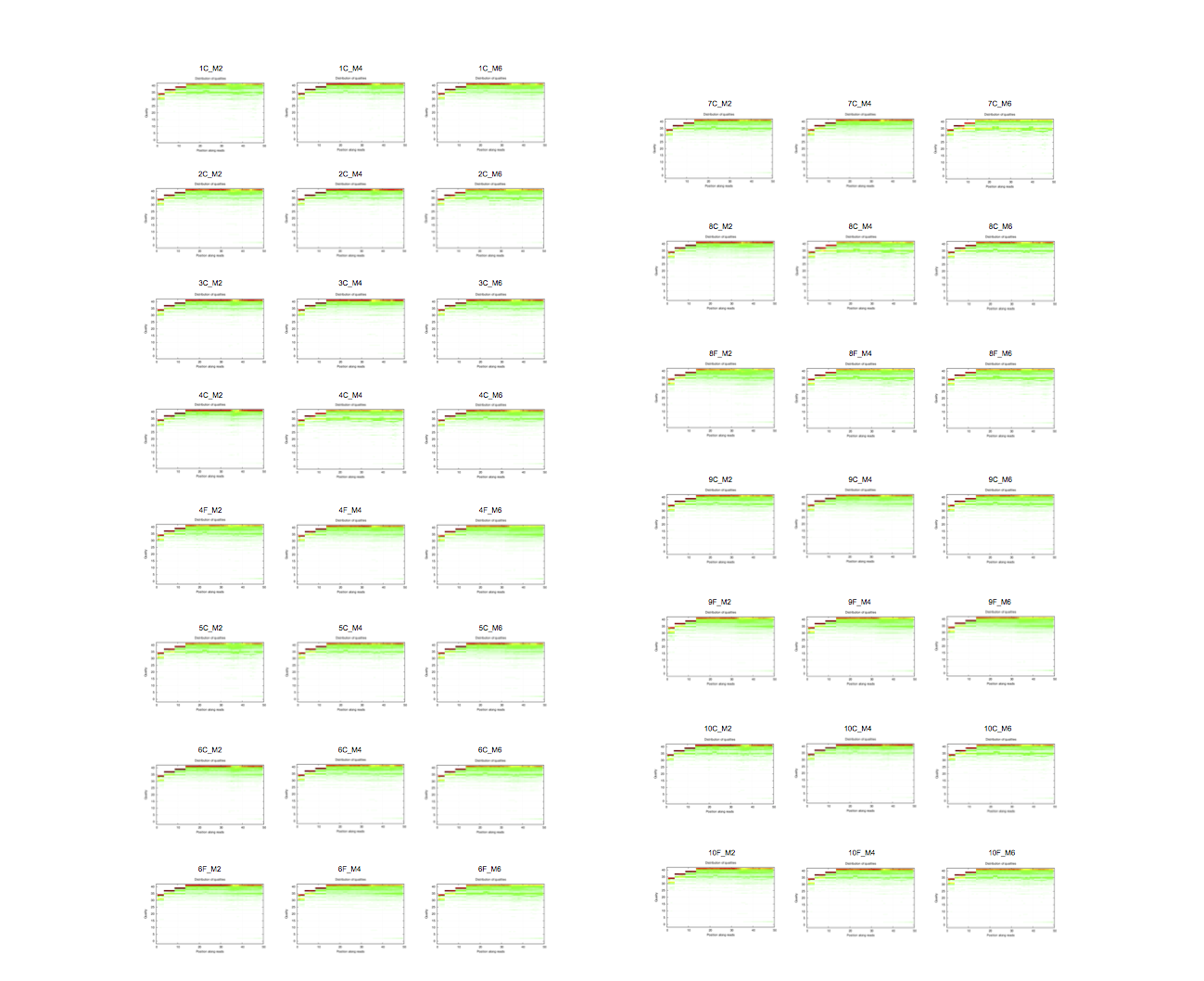

Supplement: S1 Fig — Reads quality control in each sample (n = 45) after cleaning, where miRNA length is considered between 18–24 nt. (TIFF) [file pone.0152610.s001.tiff]

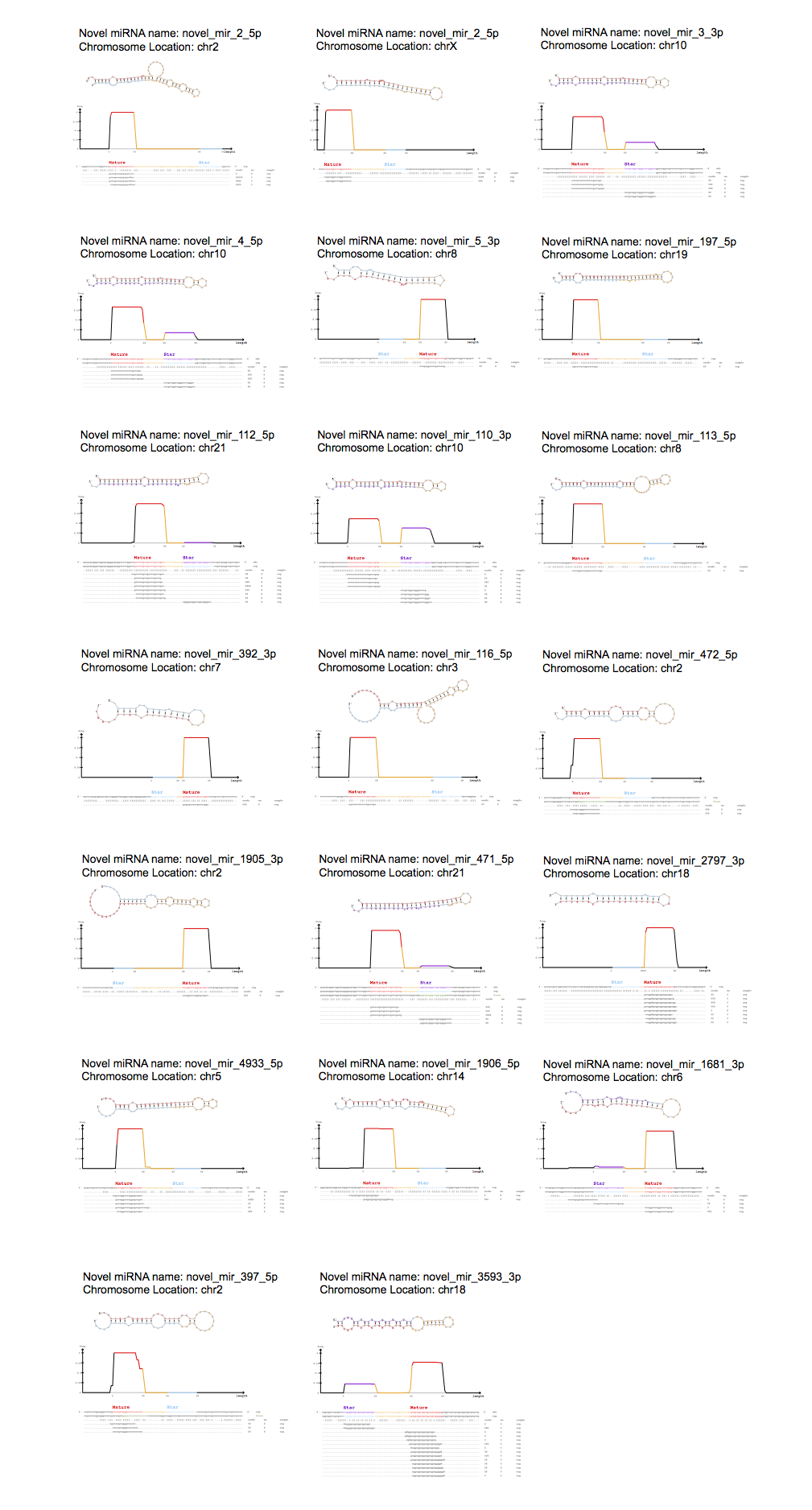

Supplement: S2 Fig — (TIFF) [file pone.0152610.s002.tiff]

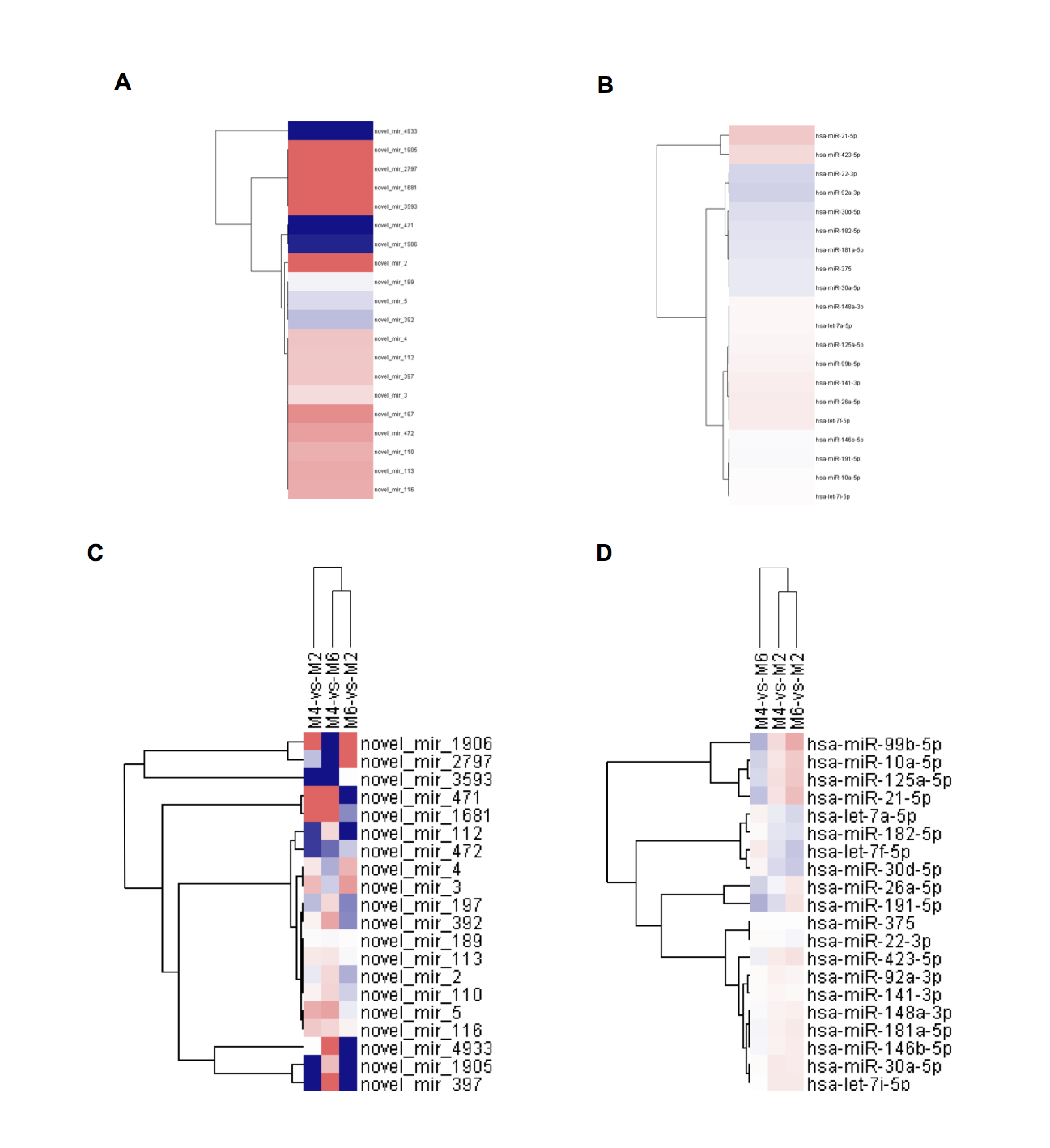

Supplement: S3 Fig — The expression levels were analysed hierarchically by clustering the top 20 miRNAs, where the blue colour shows the downregulated miRNAs, whilst the red colour shows the upregulated miRNAs. (TIFF) [file pone.0152610.s003.tiff]
